# Supplementary material for: Extreme electron–hole drag and negative mobility in the Dirac plasma of graphene
Source: Nat Commun. 2024 Nov 14;15:9869. doi: 10.1038/s41467-024-54198-x (PMC11564795; doi:10.1038/s41467-024-54198-x)
Supplement: Supplementary file 1 — Supplementary Information [file 41467_2024_54198_MOESM1_ESM.pdf]

Supplementary Information

Extreme electron-hole drag and negative mobility in the Dirac plasma of graphene

SUPPLEMENTARY NOTE 1. HALL RESISTANCE AT HIGH CARRIER DENSITIES

When $n_e \ll n_h$ or $n_h \gg n_e$, the current is dominated by the majority charge carrier and we can obtain the value of $n = n_e - n_h$ from the measured Hall resistance, R_H, in an applied magnetic field, B, using the following expression

$$\frac{n}{B} = \frac{1}{eR_H}. \tag{1}$$

Combining this expression with the capacitor equation for the graphene layer

$$n = (V_g - V_{NP})/Ce, \tag{2}$$

where $C/e = 5.88 \times 10^{10}$ cm^{-2}V^{-1} is the areal capacitance of Device 1. We obtain the following relation between $1/eR_H$ and gate voltage, V_g, when either $n \approx n_e$ or $n \approx n_h$,

$$\frac{1}{eR_H} = \frac{n}{B} = \frac{(V_g - V_{NP})C}{Be}. \tag{3}$$

This linear relation is demonstrated in the plot of $1/eR_H = n/B$ as a function of gate voltage, see Supplementary Fig. 1, from which we obtain an accurate measurement of the magnetic field, B.

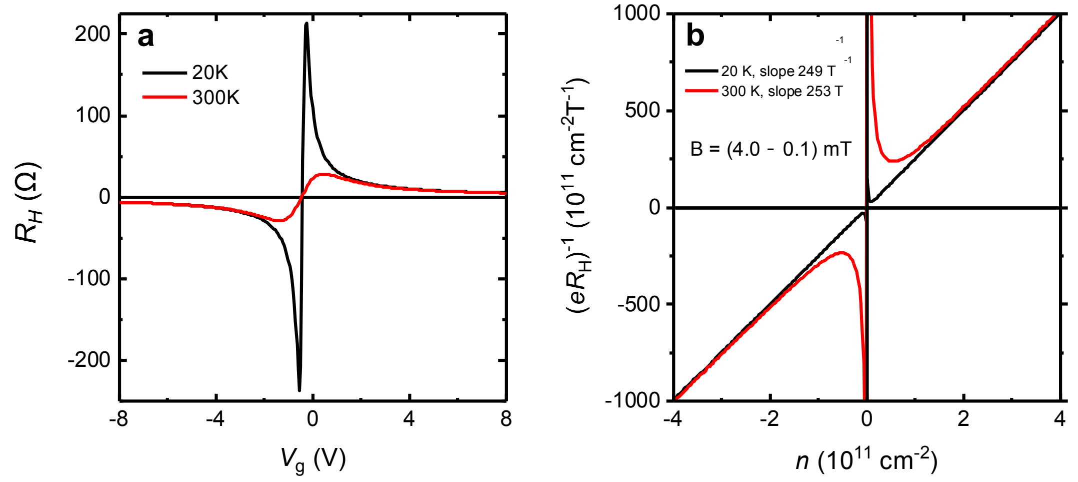

Supplementary Figure 1. (**a**) Hall resistance as a function of gate voltage measured in weak magnetic field of a few mT at 20 K and 300 K, as indicated. (**b**) Same data as in **a** presented as $1/(eR_H)$ vs n. n was extracted from Hall resistance measurements at higher field, $B = 0.1$ T. This data set allows accurate determination of magnetic field, which was kept constant within 2% during the experiment.

In our experiment, the value of B was kept below a few mT to avoid any nonlinear-in-B behavior in the Hall resistance, which becomes noticable near the neutrality point for magnetic fields above 10 mT. This also enabled us to disregard any changes in the longitudinal resistivity as magnetoresistance remained below 1% over our entire temperature range.

SUPPLEMENTARY NOTE 2. CARRIER STATISTICS

The electron and hole concentrations, n_e and n_h in the graphene layer are determined by integrating the product of the density of states of graphene $DoS(\varepsilon_{\boldsymbol{k}})$ with the Fermi-Dirac distribution function, $f^{(0)}_{\boldsymbol{k},\nu}(\varepsilon_{\boldsymbol{k}}, \Theta)$,

$$n_\nu = \int f_\nu^{(0)}(\varepsilon_{\boldsymbol{k}}, \Theta) DoS(\varepsilon_{\boldsymbol{k}}) d\varepsilon_{\boldsymbol{k}}, \tag{4}$$

where $\nu = e$ or h labels the carrier type, Θ is the chemical potential and

$$DoS\left(\varepsilon_{\boldsymbol{k}}\right) = \frac{2|\varepsilon_{\boldsymbol{k}}|}{\pi\hbar^2 v_F^2}, \quad f_{\boldsymbol{k},e}^{(0)}(\varepsilon_{\boldsymbol{k}}, \Theta) = \frac{1}{e^{(\varepsilon_{\boldsymbol{k}}-\Theta)/k_BT}+1}, \quad f_{\boldsymbol{k},h}^{(0)}(\varepsilon_{\boldsymbol{k}}, \Theta) = 1 - f_{\boldsymbol{k},e}^{(0)}(\varepsilon_{\boldsymbol{k}}, \Theta) = \frac{1}{e^{(\varepsilon_{\boldsymbol{k}}+\Theta)/k_BT}+1} \tag{5}$$

which after using the complete Fermi-Dirac integral [1] gives rise to the following expression for n_e and n_h

$$n_{e,h} = -\frac{2}{\pi}\left(\frac{k_BT}{\hbar v_F}\right)^2 Li_2[-\exp(\pm\Theta/k_BT)]. \tag{6}$$

Here $Li_n(x)$ is the polylogarithm function of the nth order, and the $+(-)$ sign corresponds to electrons and holes respectively. It is straightforward to show that when $n = \Theta = 0$,

$$n_e = n_h = n_{th} = \frac{\pi}{6}\left(\frac{k_BT}{\hbar v_F}\right)^2 = \frac{2\pi^3}{3}\left(\frac{k_BT}{hv_F}\right)^2. \tag{7}$$

We determine $\Theta(n)$ by solving numerically the following equation:

$$n = -\frac{2}{\pi}\left(\frac{k_BT}{\hbar v_F}\right)^2 \left(Li_2[-\exp(\Theta/k_BT)] - Li_2[-\exp(-\Theta/k_BT)]\right). \tag{8}$$

which we then use to obtain $n_e(n)$ and $n_h(n)$, see Supplementary Fig. 2. It is useful to work with the following dimensionless densities,

$$\eta_e = n_e\left(\frac{\hbar v_F}{k_BT}\right)^2, \ \eta_h = n_h\left(\frac{\hbar v_F}{k_BT}\right)^2 \ \text{and} \ \eta = n\left(\frac{\hbar v_F}{k_BT}\right)^2 \tag{9}$$

and dimensionless chemical potential,

$$\mathcal{T} = \Theta/k_BT. \tag{10}$$

In these units, we obtain a temperature-independent expression for η:

$$\eta = -\frac{2}{\pi}\left(Li_2[-\exp(\mathcal{T})] - Li_2[-\exp(\mathcal{T})]\right). \tag{11}$$

The Hall resistance when the mobilities of the electrons and holes are equal is given by:

$$R_H = \frac{B}{e}\frac{n}{(n_h+n_e)^2}. \tag{12}$$

If we define the dimensionless Hall resistance as

$$Y_H = \frac{eR_H}{B}\left(\frac{k_BT}{\hbar v_F}\right)^2, \tag{13}$$

we find that by inserting Eq. 12 into Eq. 13, Y_H takes the form

$$Y_H(\mathcal{T}) = \left(\frac{\pi}{2}\right)^2 \frac{\eta}{\left(Li_2(-e^{-\mathcal{T}}) + Li_2(-e^{\mathcal{T}})\right)^2}. \tag{14}$$

This expression reveals that both n and R_H scale as T^2 (in the case of equal electron and hole mobilities), so that we can collapse the curves of $R_H(n)$ measured at different temperatures onto a single universal curve.

SUPPLEMENTARY NOTE 3. TRANSPORT MODEL FOR ELECTRON-HOLE DRAG

A. The kinetic equation

In this section we derive expressions for the electron and hole mobilities in the presence of electron-hole interactions in the Dirac plasma. We start from the steady-state homogeneous kinetic equation for the distribution of *electrons* $f_{\boldsymbol{k},\lambda}$ in the conduction ($\lambda = +$) and valence ($\lambda = -$) bands in applied electric, $\boldsymbol{E}$, and magnetic, $\boldsymbol{B}$ fields

$$-e(\boldsymbol{E} + \boldsymbol{v}_{\boldsymbol{k},\lambda} \times \boldsymbol{B}) \cdot \nabla_{\boldsymbol{k}} f_{\boldsymbol{k},\lambda} = \mathcal{I}_{ee}[f_{\boldsymbol{k},\lambda}] + \mathcal{I}_{\text{dis/ph}}[f_{\boldsymbol{k},\lambda}], \tag{15}$$

where $\boldsymbol{v} = \partial\varepsilon_{\boldsymbol{k}}/\hbar\partial\boldsymbol{k}$, and $\mathcal{I}_{ee}[f_{\boldsymbol{k},\lambda}(\boldsymbol{r},t)]$ and $\mathcal{I}_{\text{dis/ph}}[f_{\boldsymbol{k},\lambda}(\boldsymbol{r},t)]$ are the collision integrals of the electron-electron and electron-disorder (or electron-phonon) interactions, respectively. Note that electron-electron interactions, although globally momentum conserving, do not conserve the momentum of particles in an individual band. To derive an expression for the electron-hole drag, we will consider only interactions between electrons in different bands that conserve the number of electrons in each band, i.e.

$$\begin{aligned}\mathcal{I}_{ee}[f_{\boldsymbol{k},\lambda}] &= -\sum_{\boldsymbol{k}_2,\boldsymbol{k}_3;\boldsymbol{k}_4} W^{\text{ee}}_{\boldsymbol{k},\lambda;\boldsymbol{k}_2,\bar{\lambda}\to\boldsymbol{k}_3,\lambda;\boldsymbol{k}_4,\bar{\lambda}}\delta(\boldsymbol{k}+\boldsymbol{k}_2-\boldsymbol{k}_3-\boldsymbol{k}_4)\delta(\varepsilon_{\boldsymbol{k},\lambda}+\varepsilon_{\boldsymbol{k}_2,\bar{\lambda}}-\varepsilon_{\boldsymbol{k}_3,\lambda}-\varepsilon_{\boldsymbol{k}_4,\bar{\lambda}})\\ &\times\left[f_{\boldsymbol{k},\lambda}f_{\boldsymbol{k}_2,\bar{\lambda}}(1-f_{\boldsymbol{k}_3,\lambda})(1-f_{\boldsymbol{k}_4,\bar{\lambda}})-(1-f_{\boldsymbol{k},\lambda})(1-f_{\boldsymbol{k}_2,\bar{\lambda}})f_{\boldsymbol{k}_3,\lambda}f_{\boldsymbol{k}_4,\bar{\lambda}}\right].\end{aligned} \tag{16}$$

Here, $W^{ee}_{\boldsymbol{k}.\lambda;\boldsymbol{k}_2,\lambda_2\to\boldsymbol{k}_3,\lambda;\boldsymbol{k}_4,\lambda}$ is the squared matrix element of the Coulomb interaction. Similarly, the disorder and phonon collision integrals are

$$\mathcal{I}_{\text{dis}}[f_{\boldsymbol{k},\lambda}] = -\sum_{\boldsymbol{k}'} W^{(\text{dis})}_{\boldsymbol{k},\lambda\to\boldsymbol{k}',\lambda}\delta(\varepsilon_{\boldsymbol{k},\lambda}-\varepsilon_{\boldsymbol{k}',\lambda})(f_{\boldsymbol{k},\lambda}-f_{\boldsymbol{k}',\lambda}). \tag{17}$$

and

$$\begin{aligned}\mathcal{I}_{\text{ph}}[f_{\boldsymbol{k},\lambda}] &= -\sum_{\boldsymbol{k}',\lambda'}\sum_{\boldsymbol{q},\nu}\Big\{W^{(\text{ph})}_{\boldsymbol{k},\lambda\to\boldsymbol{k}',\lambda'}(\boldsymbol{q},\nu)\delta(\boldsymbol{k}'-\boldsymbol{k}-\boldsymbol{q})\delta(\varepsilon_{\boldsymbol{k}',\lambda'}-\varepsilon_{\boldsymbol{k},\lambda}-\omega_{\boldsymbol{q},\nu})\\ &\times\left[f_{\boldsymbol{k},\lambda}(1-f_{\boldsymbol{k}',\lambda'})n_{\boldsymbol{q},\nu}-f_{\boldsymbol{k}',\lambda'}(1-f_{\boldsymbol{k},\lambda})(n_{\boldsymbol{q},\nu}+1)\right]\\ &-W^{(\text{ph})}_{\boldsymbol{k}',\lambda'\to\boldsymbol{k},\lambda}(\boldsymbol{q},\nu)\delta(\boldsymbol{k}-\boldsymbol{k}'-\boldsymbol{q})\delta(\varepsilon_{\boldsymbol{k},\lambda}-\varepsilon_{\boldsymbol{k}',\lambda'}-\omega_{\boldsymbol{q},\nu})\\ &\times\left[f_{\boldsymbol{k}',\lambda'}(1-f_{\boldsymbol{k}',\lambda'})n_{\boldsymbol{q},\nu}-f_{\boldsymbol{k},\lambda}(1-f_{\boldsymbol{k}',\lambda'})(n_{\boldsymbol{q},\nu}+1)\right]\Big\},\end{aligned} \tag{18}$$

Here, $\boldsymbol{q}$ is the phonon momentum, ν its polarization and $\omega_{\boldsymbol{q},\nu}$ its energy. Finally, $n_{\boldsymbol{q},\nu}$ is the phonon distribution function. Note that only intraband transitions contribute to $\mathcal{I}_{ee}[f_{\boldsymbol{k},\lambda}]$, whereas interband ones can contribute to the electron-phonon collision integral, in particular when the Fermi energy is close to the charge neutrality point. In the linear response regime, the distribution function is approximated by

$$f_{\boldsymbol{k},\lambda} \equiv f^{(0)}(\varepsilon_{\boldsymbol{k},\lambda}) + \left(-\frac{\partial f^{(0)}(\varepsilon_{\boldsymbol{k},\lambda})}{\partial\varepsilon_{\boldsymbol{k},\lambda}}\right)\boldsymbol{k}\cdot\boldsymbol{u}_\lambda, \tag{19}$$

so that Eq. 15 becomes

$$-e(\boldsymbol{E}+\boldsymbol{u}_\lambda\times\boldsymbol{B})\cdot\boldsymbol{v}_{\boldsymbol{k},\lambda}\left(-\frac{\partial f^{(0)}(\varepsilon_{\boldsymbol{k},\lambda})}{\partial\varepsilon_{\boldsymbol{k},\lambda}}\right) = -\mathcal{I}_{\text{ee}}[f_{\boldsymbol{k},\lambda}]-\mathcal{I}_{\text{dis/ph}}[f_{\boldsymbol{k},\lambda}]. \tag{20}$$

We now introduce $f_{\boldsymbol{k},e} = f_{\boldsymbol{k},+}$, $f_{\boldsymbol{k},h} = 1 - f_{-\boldsymbol{k},-}$, $\boldsymbol{u}_e = \boldsymbol{u}_+$ and $\boldsymbol{u}_h = \boldsymbol{u}_-$. We obtain the following equations for electrons and holes respectively

$$\mp e(\boldsymbol{E}+\boldsymbol{u}_\nu\times\boldsymbol{B})\cdot\boldsymbol{v}_{\boldsymbol{k}}\left(-\partial_{\varepsilon_{\boldsymbol{k}}}f^{(0)}_{\boldsymbol{k},\nu}\right) = \mathcal{I}_{\nu\bar{\nu}}+\mathcal{I}^{(\text{dis/ph})}_\nu, \tag{21}$$

where $\nu = e,h$ ($\bar{\nu} = h,e$) and the $-$ and $+$ sign is for electrons and holes respectively. We also have $f^{(0)}_{\boldsymbol{k},e} = [e^{\beta(\varepsilon_{\boldsymbol{k}}-\mu)}+1]^{-1}$ and $f^{(0)}_{\boldsymbol{k},h} = [e^{\beta(\varepsilon_{\boldsymbol{k}}+\mu)}+1]^{-1}$, so that

$$f_{\boldsymbol{k},\nu} \equiv f^{(0)}_{\boldsymbol{k},\nu} + (-\partial_{\varepsilon\boldsymbol{k}}f^{(0)}_{\boldsymbol{k},\nu})\boldsymbol{k}\cdot\boldsymbol{u}_\nu \tag{22}$$

and

$$\begin{aligned}\mathcal{I}_{\nu\bar{\nu}} \;=\; & \sum_{\boldsymbol{k}_2,\boldsymbol{k}_3,\boldsymbol{k}_4} W^{\nu\bar{\nu}}_{\boldsymbol{k};\boldsymbol{k}_2\to\boldsymbol{k}_3;\boldsymbol{k}_4}\delta(\boldsymbol{k}+\boldsymbol{k}_2-\boldsymbol{k}_3-\boldsymbol{k}_4)\delta(\varepsilon_{\boldsymbol{k}}+\varepsilon_{\boldsymbol{k}_2}-\varepsilon_{\boldsymbol{k}_3}-\varepsilon_{\boldsymbol{k}_4}) \\ & \times\left[f_{\boldsymbol{k},\nu}f_{\boldsymbol{k}_2,\bar{\nu}}(1-f_{\boldsymbol{k}_3,\nu})(1-f_{\boldsymbol{k}_4,\bar{\nu}})-(1-f_{\boldsymbol{k},\nu})(1-f_{\boldsymbol{k}_2,\bar{\nu}})f_{\boldsymbol{k}_3,\nu}f_{\boldsymbol{k}_4,\bar{\nu}}\right]. \end{aligned} \tag{23}$$

Here, $W^{eh}_{\boldsymbol{k};\boldsymbol{k}_2\to\boldsymbol{k}_3;\boldsymbol{k}_4} = W^{ee}_{+\boldsymbol{k}.+;-,\boldsymbol{k}_2,-\to+\boldsymbol{k}_3,+;-\boldsymbol{k}_4,-}$ and $W^{he}_{\boldsymbol{k};\boldsymbol{k}_2\to\boldsymbol{k}_3;\boldsymbol{k}_4} = W^{ee}_{-\boldsymbol{k}.-;+,\boldsymbol{k}_2,+\to-\boldsymbol{k}_3,-;+\boldsymbol{k}_4,+}$ and

$$\mathcal{I}^{\rm dis}_{\nu} = \sum_{\boldsymbol{k}'} W^{\rm dis}_{\boldsymbol{k}\to\boldsymbol{k}'}\delta(\varepsilon_{\boldsymbol{k}}-\varepsilon_{\boldsymbol{k}'})(f_{\boldsymbol{k},\nu}-f_{\boldsymbol{k}',\nu}) \tag{24}$$

Using Eq. 22, we can rewrite the collision integral in Eq. 23 as

$$\begin{aligned}\mathcal{I}_{\nu\bar{\nu}} \simeq & \frac{1}{k_BT}\sum_{\boldsymbol{k}_2,\boldsymbol{k}_3,\boldsymbol{k}_4} W^{\nu\bar{\nu}}_{\boldsymbol{k};\boldsymbol{k}_2\to\boldsymbol{k}_3;\boldsymbol{k}_4}\delta(\boldsymbol{k}+\boldsymbol{k}_2-\boldsymbol{k}_3-\boldsymbol{k}_4)\delta(\varepsilon_{\boldsymbol{k}}+\varepsilon_{\boldsymbol{k}_2}-\varepsilon_{\boldsymbol{k}_3}-\varepsilon_{\boldsymbol{k}_4}) \\ & \times f^{(0)}_{\boldsymbol{k},\nu}f^{(0)}_{\boldsymbol{k}_2,\bar{\nu}}(1-f^{(0)}_{\boldsymbol{k}_3,\nu})(1-f^{(0)}_{\boldsymbol{k}_4,\bar{\nu}})(\boldsymbol{k}-\boldsymbol{k}_3)\cdot(\boldsymbol{u}_\nu-\boldsymbol{u}_{\bar{\nu}}) \end{aligned} \tag{25}$$

We can verify that the collision integral in Eq. 23 conserves the total momentum, i.e. the momentum lost by the electrons is gained by the holes, and vice versa. We do this by calculating

$$\begin{aligned}\sum_{\boldsymbol{k}}\boldsymbol{k}(\mathcal{I}_{eh}+\mathcal{I}_{he}) = & \sum_{\boldsymbol{k},\boldsymbol{k}_2,\boldsymbol{k}_3,\boldsymbol{k}_4}\boldsymbol{k}W^{eh}_{\boldsymbol{k};\boldsymbol{k}_2\to\boldsymbol{k}_3;\boldsymbol{k}_4}\delta(\boldsymbol{k}+\boldsymbol{k}_2-\boldsymbol{k}_3-\boldsymbol{k}_4)\delta(\varepsilon_{\boldsymbol{k}}+\varepsilon_{\boldsymbol{k}_2}-\varepsilon_{\boldsymbol{k}_3}-\varepsilon_{\boldsymbol{k}_4}) \\ & \times\Big\{\left[f_{\boldsymbol{k},e}f_{\boldsymbol{k}_2,h}(1-f_{\boldsymbol{k}_3,e})(1-f_{\boldsymbol{k}_4,h})-(1-f_{\boldsymbol{k},e})(1-f_{\boldsymbol{k}_2,h})f_{\boldsymbol{k}_3,e}f_{\boldsymbol{k}_4,h}\right] \\ & +\;\left[f_{\boldsymbol{k},h}f_{\boldsymbol{k}_2,e}(1-f_{\boldsymbol{k}_3,h})(1-f_{\boldsymbol{k}_4,e})-(1-f_{\boldsymbol{k},h})(1-f_{\boldsymbol{k}_2,e})f_{\boldsymbol{k}_3,h}f_{\boldsymbol{k}_4,e}\right]\Big\} \\ = & \frac{1}{2}\sum_{\boldsymbol{k},\boldsymbol{k}_2,\boldsymbol{k}_3,\boldsymbol{k}_4} W^{eh}_{\boldsymbol{k};\boldsymbol{k}_2\to\boldsymbol{k}_3;\boldsymbol{k}_4}\delta(\boldsymbol{k}+\boldsymbol{k}_2-\boldsymbol{k}_3-\boldsymbol{k}_4)\delta(\varepsilon_{\boldsymbol{k}}+\varepsilon_{\boldsymbol{k}_2}-\varepsilon_{\boldsymbol{k}_3}-\varepsilon_{\boldsymbol{k}_4}) \\ & \times\Big\{(\boldsymbol{k}-\boldsymbol{k}_3)\left[f_{\boldsymbol{k},e}f_{\boldsymbol{k}_2,h}(1-f_{\boldsymbol{k}_3,e})(1-f_{\boldsymbol{k}_4,h})-(1-f_{\boldsymbol{k},e})(1-f_{\boldsymbol{k}_2,e})f_{\boldsymbol{k}_3,e}f_{\boldsymbol{k}_4,h}\right] \\ & +\;(\boldsymbol{k}-\boldsymbol{k}_4)\left[f_{\boldsymbol{k},h}f_{\boldsymbol{k}_2,e}(1-f_{\boldsymbol{k}_3,h})(1-f_{\boldsymbol{k}_4,e})-(1-f_{\boldsymbol{k},h})(1-f_{\boldsymbol{k}_2,e})f_{\boldsymbol{k}_3,h}f_{\boldsymbol{k}_4,e}\right]\Big\} \\ = & \frac{1}{2}\sum_{\boldsymbol{k},\boldsymbol{k}_2,\boldsymbol{k}_3,\boldsymbol{k}_4} W^{eh}_{\boldsymbol{k};\boldsymbol{k}_2\to\boldsymbol{k}_3;\boldsymbol{k}_4}\delta(\boldsymbol{k}+\boldsymbol{k}_2-\boldsymbol{k}_3-\boldsymbol{k}_4)\delta(\varepsilon_{\boldsymbol{k}}+\varepsilon_{\boldsymbol{k}_2}-\varepsilon_{\boldsymbol{k}_3}-\varepsilon_{\boldsymbol{k}_4}) \\ & \times\Big\{(\boldsymbol{k}-\boldsymbol{k}_3+\boldsymbol{k}_2-\boldsymbol{k}_4)\left[f_{\boldsymbol{k},e}f_{\boldsymbol{k}_2,h}(1-f_{\boldsymbol{k}_3,e})(1-f_{\boldsymbol{k}_4,h})-(1-f_{\boldsymbol{k},e})(1-f_{\boldsymbol{k}_2,h})f_{\boldsymbol{k}_3,e}f_{\boldsymbol{k}_4,h}\right] \\ = & 0, \end{aligned} \tag{26}$$

as required.

B. Relaxation time approximation

We parameterise the collision integrals using the relaxation time approximation. Collisions between electrons and holes or with lattice disorder or impurities and phonons tend to relax the respective particle distributions towards equilibrium. Therefore, within the relaxation-time approximation, we can write

$$\mathcal{I}^{\rm dis/ph}_{\nu} \simeq \frac{f_{\boldsymbol{k},\nu}-f^{(0)}(\varepsilon_{\boldsymbol{k},\nu})}{\tau} = (-\partial_{\varepsilon_{\boldsymbol{k}}}f^{(0)}_{\boldsymbol{k},\nu})\frac{\boldsymbol{k}\cdot\boldsymbol{u}_\nu}{\tau}\;. \tag{27}$$

Conversely, collisions between electrons and holes relax their respective distributions to a distribution function characterised by a single drift velocity $\boldsymbol{u}$, i.e.

$$\bar{f}_{\boldsymbol{k},\nu} \;\equiv\; f^{(0)}_{\boldsymbol{k},\nu}+(-\partial_{\varepsilon_{\boldsymbol{k}}}f^{(0)}_{\boldsymbol{k},\nu})\boldsymbol{k}\cdot\boldsymbol{u}\;. \tag{28}$$

Therefore, within a "generalized" relaxation-time approximation,

$$\mathcal{I}_{\nu\bar{\nu}} \simeq \frac{f_{\boldsymbol{k},\nu}-\bar{f}_{\boldsymbol{k},\nu}}{\tau_{eh}} = (-\partial_{\varepsilon_{\boldsymbol{k}}}f^{(0)}_{\boldsymbol{k},\nu})\frac{\boldsymbol{k}\cdot(\boldsymbol{u}_\nu-\boldsymbol{u})}{\tau_{eh}}\;. \tag{29}$$

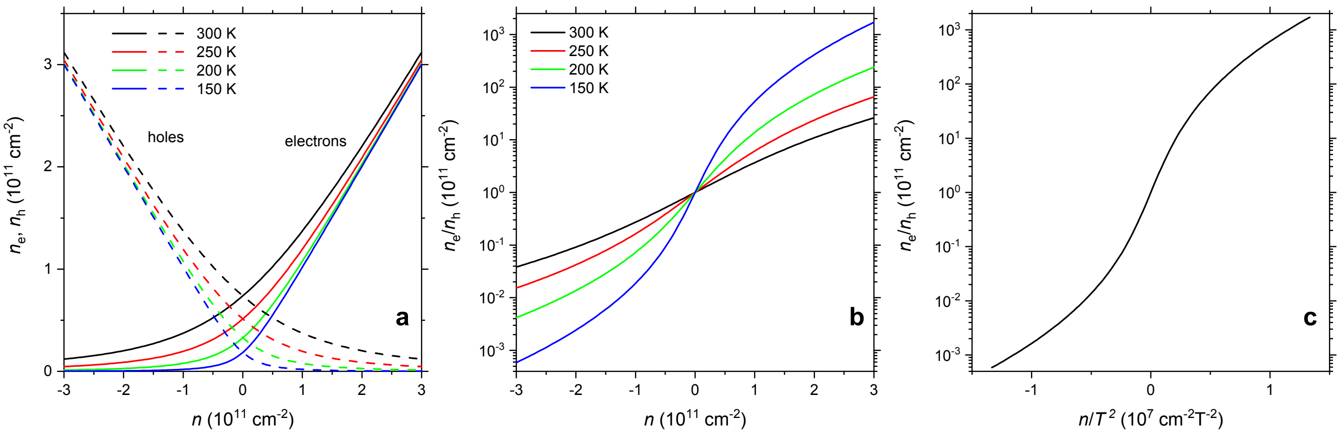

Supplementary Figure 2. (**a**) The density of electrons (n_e) and holes (n_h), calculated using Eq. 6 and plotted for various temperatures as a function of the total carrier density $n = n_e - n_h$. (**b**) the ratio between n_e and n_h. (**c**) Same as Panel (**b**), but with the total carrier density scaled with T^2. All curves collapse onto a single one.

To determine the total drift velocity $\boldsymbol{u}$ in terms of the drift velocities of electrons and holes, we assume that, within linear response:

$$\boldsymbol{u} = A_e \boldsymbol{u}_e + A_h \boldsymbol{u}_h. \tag{30}$$

We impose the conservation of total momentum by multiplying Eq. 29 by $\boldsymbol{k}$ and summing over all momenta requiring the result to be equal to zero. We thereby obtain

$$0 \;=\; \sum_\nu \frac{\boldsymbol{u}_\nu - \boldsymbol{u}}{2\tau_{eh}} \sum_{\boldsymbol{k}} (-\partial_{\varepsilon_{\boldsymbol{k}}} f^{(0)}_{\boldsymbol{k},\nu}) |\boldsymbol{k}|^2 = -\sum_{\nu=e,h} \rho_\nu \frac{\boldsymbol{u}_\nu - \boldsymbol{u}}{\tau_{eh}} \tag{31}$$

$$= -\frac{\rho_e[(1-A_e)\boldsymbol{u}_e - A_h \boldsymbol{u}_h] + \rho_h[(1-A_h)\boldsymbol{u}_h - A_e \boldsymbol{u}_e]}{\tau_{eh}}\;, \tag{32}$$

where

$$\rho_\nu \equiv \frac{1}{2}\int_0^\infty d\varepsilon \frac{\nu(\varepsilon)}{4k_{\rm B}T\cosh\left(\frac{\varepsilon\mp\Theta}{2k_{\rm B}T}\right)} \frac{\varepsilon^2}{(\hbar v_{\rm F})^2} = -3\frac{N_{\rm f}(k_{\rm B}T)^3}{2\pi(\hbar v_{\rm F}^2)^2} {\rm Li}_3\left(-e^{\pm\Theta/(k_{\rm B}T)}\right)\;, \tag{33}$$

are the mass densities of electrons (+) and holes (-), respectively. Therefore since Eq. 32 must be equal to 0 for any $\boldsymbol{u}_\nu$,

$$\rho_\nu(1-A_\nu) - \rho_{\bar\nu} A_\nu = 0 \quad\Rightarrow\quad A_\nu = \frac{\rho_\nu}{\rho_e+\rho_h}\;. \tag{34}$$

Using these results, Eq. 29 becomes

$$\mathcal{I}_{\nu\bar\nu} = \frac{\rho_{\bar\nu}}{\rho_e+\rho_h}\big(-\partial_{\varepsilon_{\boldsymbol{k}}} f^{(0)}_{\boldsymbol{k},\nu}\big)\frac{\boldsymbol{k}\cdot(\boldsymbol{u}_\nu - \boldsymbol{u}_{\bar\nu})}{\tau_{eh}}\;. \tag{35}$$

Note that the behaviour of the approximate collision integrals in Eq. 35 matches that of Eq. 25, in particular the momentum transferred between the two subsystems vanishes if the electrons and holes have the same drift velocity. Combining Eq. 19, 27 and 35 we can write the kinetic equation 15 as

$$\mp e(\boldsymbol{E} + \boldsymbol{u}_\nu\times\boldsymbol{B})\cdot\boldsymbol{v}_{\boldsymbol{k}}(-\partial_{\varepsilon_{\boldsymbol{k}}} f^{(0)}_{\boldsymbol{k},\nu}) = (-\partial_{\varepsilon_{\boldsymbol{k}}} f^{(0)}_{\boldsymbol{k},\nu})\left[\frac{\rho_{\bar\nu}}{\rho_e+\rho_h}\frac{\boldsymbol{k}\cdot(\boldsymbol{u}_\nu - \boldsymbol{u}_{\bar\nu})}{\tau_{eh}} + \frac{\boldsymbol{k}\cdot\boldsymbol{u}_\nu}{\tau}\right]\;. \tag{36}$$

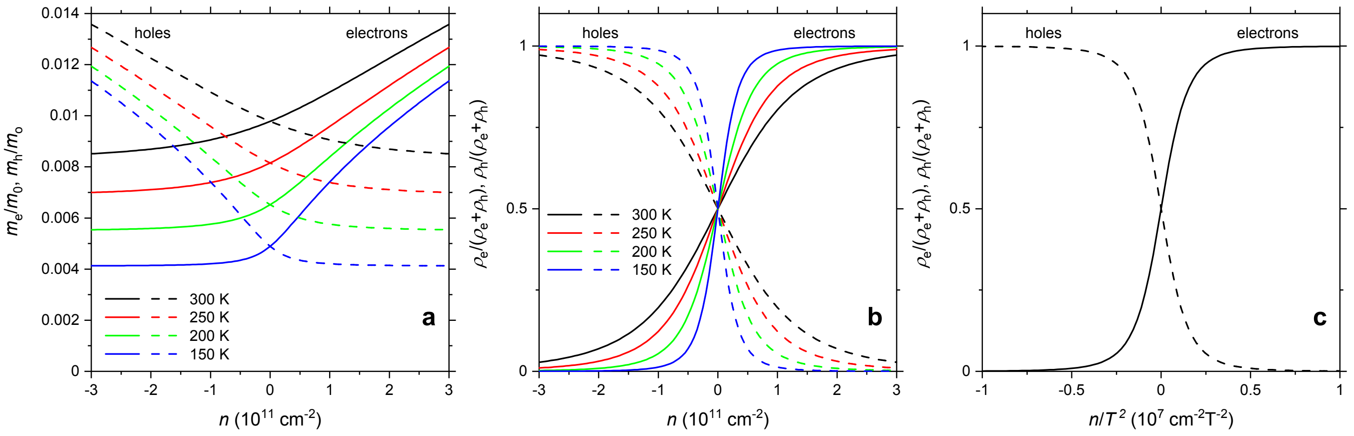

Supplementary Figure 3. (**a**) The masses of electrons (m_e) and holes (m_h), calculated using Eq. 40 and plotted for various temperatures and in units of the bare electron mass m_0 as a function of the total carrier density n. (**b**) the ratios $\rho_e/(\rho_e+\rho_h)$ and $\rho_h/(\rho_e+\rho_h)$, where ρ_e and ρ_h are the mass densities of electrons and holes, respectively, plotted for various temperatures as a function of the total carrier density n. (**c**) Same as Panel (**b**), but with the total carrier density scaled with T^2. All curves collapse onto a single one.

Multiplying this equation by $\boldsymbol{v}_{\boldsymbol{k}} = \partial_{\boldsymbol{k}}\varepsilon_{\boldsymbol{k}}/\hbar$ and summing over $\boldsymbol{k}$ we obtain

$$\mp D_\nu e(\boldsymbol{E} + \boldsymbol{u}_\nu \times \boldsymbol{B}) = \frac{n_\nu \rho_{\bar{\nu}}}{\rho_e + \rho_h} \frac{\boldsymbol{u}_\nu - \boldsymbol{u}_{\bar{\nu}}}{\tau_{eh}} + n_\nu \frac{\boldsymbol{u}_\nu}{\tau} \ , \tag{37}$$

where

$$D_\nu \ \equiv \ \frac{v_{\rm F}^2}{2} \int_0^\infty d\varepsilon \frac{\nu(\varepsilon)}{4 k_{\rm B} T \cosh^2\left(\frac{\varepsilon \mp \Theta}{2 k_{\rm B} T}\right)} \tag{38}$$

and we note that the carrier density can be expressed by

$$n_\nu \ \equiv \ \frac{1}{2} \sum_{\boldsymbol{k}} (-\partial_{\varepsilon_{\boldsymbol{k}}} f^{(0)}_{\boldsymbol{k},\nu}) \boldsymbol{k} \cdot \boldsymbol{v}_{\boldsymbol{k},\nu} = -\frac{1}{2} \sum_{\boldsymbol{k}} \boldsymbol{k} \cdot \nabla_{\boldsymbol{k}} f^{(0)}_{\boldsymbol{k},\nu} = \sum_{\boldsymbol{k}} f^{(0)}_{\boldsymbol{k},\nu} \ . \tag{39}$$

We now introduce the effective mass of electrons (+) and holes (-) respectively (Supplementary Fig. 3a),

$$\frac{1}{m_\nu} = \frac{D_\nu}{n_\nu} = -\frac{v_{\rm F}^2}{2 k_{\rm B} T} \frac{\ln\left(1 + e^{\pm\Theta/(k_{\rm B}T)}\right)}{{\rm Li}_2\left(-e^{\pm\Theta/(k_{\rm B}T)}\right)} \ , \tag{40}$$

which allows us to rewrite equation 37 as the the following coupled Drude-like formula for the drift velocity of electrons:

$$-e(\boldsymbol{E} + \boldsymbol{u}_e \times \boldsymbol{B}) - \frac{m_e \boldsymbol{u}_e}{\tau} - \frac{m_e \gamma_{eh}}{\tau_{eh}} (\boldsymbol{u}_e - \boldsymbol{u}_h) = 0 \tag{41}$$

and holes

$$e(\boldsymbol{E} + \boldsymbol{u}_h \times \boldsymbol{B}) - \frac{m_h \boldsymbol{u}_h}{\tau} - \frac{m_h \gamma_{he}}{\tau_{eh}} (\boldsymbol{u}_h - \boldsymbol{u}_e) = 0 \tag{42}$$

where (Supplementary Fig. 3b)

$$\gamma_{eh,he} = \frac{\rho_{h,e}}{\rho_e + \rho_h}. \tag{43}$$

We can rewrite these expressions in the form:

$$-\mu_{e0}(\boldsymbol{E}+\boldsymbol{u}_e\times\boldsymbol{B})-\boldsymbol{u}_e-\frac{\tau}{\tau_{eh}}\gamma_{eh}\left(\boldsymbol{u}_e-\boldsymbol{u}_h\right)=0 \tag{44}$$

and

$$\mu_{h0}(\boldsymbol{E}+\boldsymbol{u}_h\times\boldsymbol{B})-\boldsymbol{u}_h-\frac{\tau}{\tau_{eh}}\gamma_{he}\left(\boldsymbol{u}_h-\boldsymbol{u}_e\right)=0 \tag{45}$$

where we define the conventional mobility as $\mu_{\nu,0}=e\tau/m_\nu$. These expressions show explicitly that in the limit where the scattering rate $1/\tau_{eh}\to 0$ the equations are decoupled.

C. Derivation of R_H

We can solve the simultaneous equations 44 and 45 in the Hall regime with an electric field $\boldsymbol{E}=(E_x,0,0)$ and a magnetic field $\boldsymbol{B}=(0,0,B)$ applied perpendicular to the plane. As in the experiment, we consider the limit of small magnetic fields where ρ_{xx} is approximately constant, and $\rho_{xy}\ll\rho_{xx}$, see Supplementary Note 1. In this case, we obtain to first order in B, for the x component of the electron and hole drift velocities:

$$u_e^x=\frac{\alpha_e\mu_{h0}-\alpha_h\mu_{e0}-\mu_{e0}}{1+\alpha_e+\alpha_h}E_x=-\mu_{ex}E_x \tag{46}$$

and

$$u_h^x=\frac{\alpha_e\mu_{h0}-\alpha_h\mu_{e0}+\mu_{h0}}{1+\alpha_e+\alpha_h}E_x=\mu_{hx}E_x \tag{47}$$

where $\alpha_{e,h}=\gamma_{eh,he}\tau/\tau_{eh}$ and we have defined the mobility $\mu_{\nu x}$, so that $u_x=\mu_x E_x$. Assuming the dimensionless parameter $\max(|\mu_{ex}B|,|\mu_{hx}B|)$ to be much smaller than one, we can solve Eqs. 44 and 45 for the drift velocity of electrons and holes in the y direction perturbatively to first order in B. Note that higher-order corrections to the transverse velocities scale as $\left[\max(|\mu_{ex}B|,|\mu_{hx}B|)\right]^2$ which, for the parameters used in experiments, is always smaller that $\approx 10^{-2}$. For the electrons, we obtain:

$$u_e^y=-BE_x\frac{\mu_{e0}^2(1+\alpha_h)^2-\mu_{e0}\mu_{h0}(\alpha_e+2\alpha_e\alpha_h)+\mu_{h0}^2(\alpha_e+\alpha_e^2)}{(1+\alpha_e+\alpha_h)^2} \tag{48}$$

and for the holes

$$u_h^y=-BE_x\frac{\mu_{h0}^2(1+\alpha_e)^2-\mu_{e0}\mu_{h0}(\alpha_h+2\alpha_e\alpha_h)+\mu_{e0}^2(\alpha_h+\alpha_h^2)}{(1+\alpha_e+\alpha_h)^2} \tag{49}$$

We can combine with terms that are also in $\mu_{\nu x}^2$ to obtain :

$$u_e^y=-BE_x\left(\mu_{ex}^2+\alpha_e\frac{\mu_{e0}\mu_{h0}+\mu_{h0}^2}{(1+\alpha_e+\alpha_h)^2}\right)=-BE_x\mu_{eH}^2 \tag{50}$$

$$u_h^y=-BE_x\left(\mu_{hx}^2+\alpha_h\frac{\mu_{e0}\mu_{h0}+\mu_{e0}^2}{(1+\alpha_e+\alpha_h)^2}\right)=-BE_x\mu_{hH}^2 \tag{51}$$

in which we define a "Hall mobility", $\mu_{\nu H}$. To derive expressions for the conductivity, σ, we note that the total current density is given by

$$\boldsymbol{j}=-en_e\boldsymbol{u}_e+en_h\boldsymbol{u}_h=\sigma\boldsymbol{E} \tag{52}$$

Therefore we define the components of the conductivity tensor:

$$\sigma_{xx}=\frac{j_x}{E_x}=en_e\mu_{ex}+en_h\mu_{hx} \tag{53}$$

and

$$\sigma_{yx} = \frac{j_y}{E_x} = eB\left(n_e\mu_{eH}^2 - n_h\mu_{hH}^2\right) \tag{54}$$

It follows that the resistivity is given by

$$\rho = \frac{1}{en_e\mu_{ex} + en_h\mu_{hx}} \tag{55}$$

and the Hall resistivity is

$$R_H = \frac{\sigma_{yx}}{\sigma_{xx}^2} = \frac{B\left(n_e\mu_{eH}^2 - n_h\mu_{hH}^2\right)}{e\left(n_e\mu_{ex} + n_h\mu_{hx}\right)^2} \tag{56}$$

where the mobilities are

$$\mu_{ex} = \frac{\mu_{e0} - \alpha_e\mu_{h0} + \alpha_h\mu_{e0}}{1 + \alpha_e + \alpha_h}\ , \tag{57}$$

$$\mu_{hx} = \frac{\mu_{h0} + \alpha_e\mu_{h0} - \alpha_h\mu_{e0}}{1 + \alpha_e + \alpha_h}\ , \tag{58}$$

$$\mu_{eH}^2 = \mu_{ex}^2 + \alpha_e\frac{\mu_{e0}\mu_{h0} + \mu_{h0}^2}{(1 + \alpha_e + \alpha_h)^2} \tag{59}$$

$$\mu_{hH}^2 = \mu_{hx}^2 + \alpha_h\frac{\mu_{e0}\mu_{h0} + \mu_{e0}^2}{(1 + \alpha_e + \alpha_h)^2} \tag{60}$$

Using these equations we obtain the following expression:

$$R_H = \frac{B}{e}\frac{1}{(\mu_{ex}n_e + \mu_{hx}n_h)^2}\left(n_e\frac{\mu_{ex}\mu_{e0}(\alpha_h + 1) + \alpha_e\mu_{hx}\mu_{h0}}{1 + \alpha_e + \alpha_h} - n_h\frac{\mu_{hx}\mu_{h0}(\alpha_e + 1) + \alpha_h\mu_{ex}\mu_{e0}}{1 + \alpha_e + \alpha_h}\right) \tag{61}$$

The set of equations 56 to 60 provide expressions for R_H and ρ that depend on two unknowns, τ and τ_{eh} (through $\alpha_{eh,he}$). We therefore use these expressions to extract τ and τ_{eh} from the measured data. The results of this process are shown in Fig. 4a and b of the main text.

By inserting the formula for $\mu_{\nu 0}$ and $\alpha_{\nu\bar{\nu}}$ into the expressions for the mobilities, we obtain the full expressions

$$\mu_{ex} = \frac{e\tau}{m_e}\left(1 - \frac{\tau}{\tau + \tau_{eh}}\frac{m_e + m_h}{m_h}\frac{\rho_h}{\rho_e + \rho_h}\right) \tag{62}$$

$$\mu_{hx} = \frac{e\tau}{m_h}\left(1 - \frac{\tau}{\tau + \tau_{eh}}\frac{m_e + m_h}{m_e}\frac{\rho_e}{\rho_e + \rho_h}\right) \tag{63}$$

where we note that in the limit that $\tau_{eh} \to \infty$ then $\mu_{ex} \to \mu_0$. Equivalently we can obtain expressions for $\mu_{\nu H}^2$ given by

$$\mu_{eH}^2 = \mu_{ex}^2 + \tau\tau_{eh}\frac{\tau^2}{(\tau + \tau_{eh})^2}\frac{e^2}{m_h m_e}\frac{m_e + m_h}{m_h}\frac{\rho_h}{\rho_e + \rho_h} \tag{64}$$

$$\mu_{hH}^2 = \mu_{hx}^2 + \tau\tau_{eh}\frac{\tau^2}{(\tau + \tau_{eh})^2}\frac{e^2}{m_h m_e}\frac{m_e + m_h}{m_e}\frac{\rho_e}{\rho_e + \rho_h}. \tag{65}$$

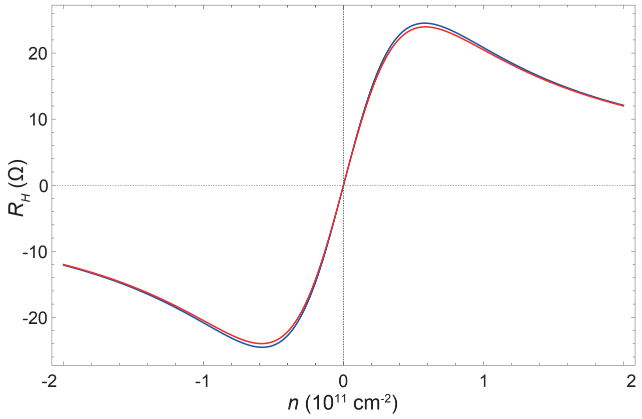

Supplementary Figure 4. The Hall resistance at 300 K calculated using the full Boltzmann model Eq. 56 (blue) is compared with the simplified expression, Eq. 66 (red). The latter has the same form as the two fluid model described in Eqs. 2 and 3 of the maintext. The close agreement of the curves validates the method of extracting the mobilities using Eq. 4 of the maintext. Here $\tau = 1.4$ ps, $\tau_{eh} = 0.35$ ps and $B = 4$ mT.

These formulae show that when the second term of the right hand side of Eqs. 64 and 65 is small, for example if $\tau_{eh} \to 0$ or $\tau \to 0$ (in which case $\mu^2 B^2 \ll 1$), then $\mu_{\nu H} \approx \mu_{\nu x}$ in which case we approximate equation 56 as

$$R_H \approx \frac{B\left(n_e \mu_{ex}^2 - n_h \mu_{hx}^2\right)}{e\left(n_e \mu_{ex} + n_h \mu_{hx}\right)^2}. \tag{66}$$

Equation 66 has the same form as that which would be derived from the Drude model as described in equations (2) and (3) of the main text. In Supplementary Fig. 4 we show the comparison of the Hall resistance calculated using the full, Eq. 56 (blue), and approximate forms Eq. 66 (red) with $\tau = 1.4$ ps and $\tau_{eh} = 0.35$ ps at 300 K. These scattering times are in close agreement with those extracted using the full expression, as shown in Fig. 4a and b of the main text. The curves in Supplementary Fig. 4 are in good agreement, validating the method of extracting the mobility given by equation 4 of the main text (with results shown in Fig. 3 of the main text).

D. Comparison with experiment

Supplementary Fig. 5a compares R_H and ρ_{xx} evaluated using Eq. 61 and Eq. 55, respectively, with our experimental data. The best fits are obtained for $\tau = 1.27$ ps and $\tau_{eh} = 0.33$ ps so that the ratio $\tau/\tau_{eh} = 3.85$. Excellent quantitative agreement is found for both $\rho_{xx}(n)$ and $R_H(n)$. To emphasize how sensitive the measurements are to the ratio τ/τ_{eh}, the figure also shows the theory curves with the ratio equal to 3 (red) and 5 (green). As the ratio decreases, the effect of electron-hole drag decreases, as expected, so that in the limit of weak electron-hole coupling the calculated curves approach the Drude model result with equal mobilities, shown as the green curve in Fig. 1c of the main text. In Supplementary Fig. 5b we show R_H measured at different T and the corresponding best fits. They yield the values of τ/τ_{eh} shown in the legend. Again, there is excellent agreement between the experimental and theoretical curves. It is only at the lowest T of 150 K, where notable deviations appear because the R_H curve is slightly asymmetric. This asymmetry is attributed to the presence of positively-charged impurities, which follows from remnant electron doping found at zero gate voltage. This is expected to break down the scattering symmetry, which, in principle, can be accounted for in our model using different values of τ for electrons and holes.

The extracted ratios τ/τ_{eh} show that, close to room temperature, the coupling between the electron and hole sub-systems in charge-neutral graphene is essential and not destroyed by scattering on phonons and impurities in high-quality devices. The extracted τ_{eh} corresponds to the quantum-critical rate,

$$\tau_{eh} = \frac{h}{C k_B T}. \tag{67}$$

where we have found $C \approx 0.6$, in good agreement with the values reported previously for both experiment and theory [3–5].

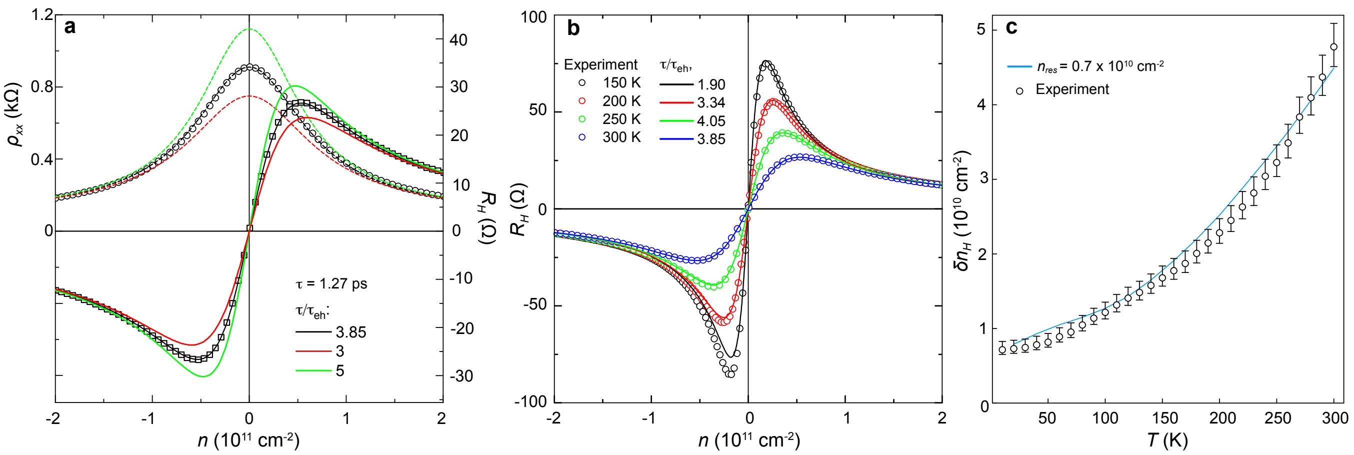

Supplementary Figure 5. (**a**) $\rho_{xx}(n)$, left hand axis, and R_H(n), right hand axis, at 300 K. Symbols: our measurements; solid (dashed) curves: R_H(n) ($\rho_{xx}(n)$) using different ratios τ/τ_{eh} (color coded). (**b**) Measured and calculated $R_H(n)$ for T between 150 and 300 K, values of τ/τ_{eh} shown in the legend. (**c**) $\delta n_H(T)$ measured (markers) and modelled (solid curve) with $n_{res} = 0.7 \times 10^{10}$ cm^{-2}. In all panels $B = 4$ mT.

E. Connection to ambipolar transport in semiconductors

The behaviour observed in this work shares conceptual similarities, but also important differences, with the phenomenon of "negative ambipolar mobility" observed in excited carriers in semiconductors. This effect, observed in GaAs quantum wells [6, 7], is beyond the conventional theory of ambipolar transport in semiconductors [8]. According to this theory, majority carriers are forced to drift along with minority carriers, unable to separate beyond a critical distance, to minimize Coulomb energy from the non-uniform charge distribution. Negative mobility, *i.e.*, the drag of minority carriers by majority carriers, first observed in Refs. [6, 7], was attributed to previously unaccounted-for frequent electron-hole scattering, and quantified through electron-hole drag trans-resistivity [7]. This trans-resistivity can be expressed in terms of an electron-hole scattering rate, as is done in our work. We note a related conceptual similarity with our study, where the standard Drude model fails to fit the experimental Hall resistivity data [Fig. 1(c) of the main text]. As in Refs. [6, 7], here too, strong electron-hole scattering must be taken into account to reproduce experimental results.

Our work presents however an extreme form of negative ambipolar effect. This strong drag effect is not typically expected in conventional semiconductors, where interactions between carriers do not modify the effective transport coefficients to the extent observed here. In graphene, due to its high purity, inter-particle interactions play a much larger role compared to conventional semiconductors, though impurity and phonon scattering still contribute. Furthermore, the symmetry between electrons and holes in graphene allows for more efficient momentum transfer between carriers, unlike in conventional semiconductors, where differing effective masses lead to weaker momentum transfer and to a less pronounced drag effect. The asymmetry in effective masses in typical semiconductors also results in significantly higher electron mobility relative to hole mobility, reducing the role of holes in transport measurements. Finally, our work benefits from a high degree of control over the carrier density in graphene, enabling us to tune the strength of the drag effect and explore this regime in detail. This control allows us to extract model parameters with a high degree of accuracy.

SUPPLEMENTARY NOTE 4. EFFECT OF ELECTRON-HOLE PUDDLES

In this section we consider effect of charge inhomogeneity ("electron-hole puddles"). In our device we estimate the residual charge density $n_{res} \sim 5 \times 10^9$ cm^{-2} (see main text). When the thermally excited carrier density, n_{th} is much larger than n_{res} ($T \gtrsim 150$ K) the effects of charge inhomogeneity can be neglected. In the following section we show that we can also model the measured data when $n_{th} \sim n_{res}$ ($T \lesssim 100$ K) by using a simple model that combines the effects of charge inhomogeneity with the electron-hole drag model of Supplementary Note 3.

A. Model for electron-hole puddles

To determine the effect of electron-hole puddles on the electronic properties of our graphene layer we employ a simple model which reproduces accurately the key characteristics of the temperature dependence of the Hall resistance. The model is based on an inhomogeneous system that has two regions $r = 1$ and 2 with different values of n_r, $n_1 = n + n_{res}$ and $n_2 = n - n_{res}$, where n_{res} is the residual carrier density associated with the inhomogeneities in the graphene layer. These two regions have electron and hole densities $n_{\nu,r}$; mobilities, $\mu_{\nu,r}$; and conductivities σ_r. It was shown in Ref. [2] that the effective conductivity tensor for this system is given by:

$$\sigma = \frac{\sigma_0^*}{1+\beta^{*2}} \begin{pmatrix} 1 & -\beta^* \\ \beta^* & 1 \end{pmatrix} \tag{68}$$

where

$$\sigma_0^* = \left(\frac{\sigma_1 \sigma_2}{1 + (\sigma_1 \beta_2 - \sigma_2 \beta_1)^2 / (\sigma_1 + \sigma_2)^2} \right)^{1/2}, \tag{69}$$

$$\beta_r = \frac{B(\mu_{ex,r}^2 n_{e,r} - \mu_{hx,r}^2 n_{h,r})}{(\mu_{ex,r} n_{e,r} + \mu_{hx,r} n_{h,r})}, \tag{70}$$

$$\sigma_r = e\left(\mu_{ex,r} n_{e,r} + \mu_{hx,r} n_{h,r}\right) \tag{71}$$

and

$$\beta^* = \sigma^* \frac{\beta_1 + \beta_2}{\sigma_1 + \sigma_2}. \tag{72}$$

Hence, the Hall resistivity is given by

$$R_H = \frac{\beta^*}{\sigma_0^*} = \frac{\beta_1 + \beta_2}{\sigma_1 + \sigma_2}. \tag{73}$$

B. Puddles in the model without electron-hole scattering

Eq. 73 can be used to evaluate how charge inhomogeneities affect predictions of the standard Drude model discussed in the main text and Section 2 above. The primary question is whether inhomogeneities alone can account for the deviation of the experimentally measured width δn_H (symbols in Fig. 2b of the main text) from the theoretical curve shown by the blue dashed line. Intuitively, the answer appears to be no, as the theory predicts broader regions between the two extrema in the $R_H(n)$ curves than what is experimentally observed. Inhomogeneities can only widen these regions (that is, increase δn_H), not narrow them. Eq. 73 provides a quantitative means to demonstrate this. Using $n_{\text{res}} = 5 \times 10^{10}\text{cm}^{-2}$, we can simulate $R_H(n)$ curves with δn_H that closely match the measured ones at low temperatures (solid blue line in Fig. 2b). However, at higher temperatures (above 100 K) where the contribution from thermally excited carriers dominates over that from electron-hole puddles, the simplified model, with or without puddles, yields practically identical values.

C. Temperature dependence of δn_H

In order to model the Hall data over a wider set of temperatures we use a simple approximation for the temperature dependence of τ/τ_{eh}. We set a constant scattering time $\tau = 2$ ps and define τ_{eh} using Eq. 67 so that the ratio of τ/τ_{eh} varies linearly with temperature and ranges from 0 to ~ 5, for T between 0 and 300 K. This approximates the dependence of τ/τ_{eh} which is shown in Fig. 4b of the main text. We then use these expressions for τ/τ_{eh} to determine the mobilities in the electron-hole puddle expression for R_H, see Eq. 73.

We find our model can explain the sharp transitions in R_H around the NP, as described by δn_H, within the experimental error over the full temperature range, see Supplementary Fig. 5c. At low temperatures, δn_H is dominated by electron hole puddles and, if we use the model presented in supplementary note 4A, both the electron-hole drag Boltzmann model and the simple Drude model (see blue curve in Fig. 2b of the main text) can explain the measured

data in this regime. However, at higher temperatures, as more electrons and holes are thermally excited and the effects of electron-hole drag become more important, the full Boltzmann model provides much closer agreement to the measured $\delta n_H(T)$ compared to the non-interacting Drude model (blue curve in Fig. 2b of the main text) which significantly over estimates δn_H. Close to room temperature, the modelled and measured curves start to deivate slightly, as expected since the extracted ratio τ/τ_{eh} starts to saturate at ~ 230 K, see inset of Fig. 4b of the main text. This is likely due to phonon scattering becoming more significant for the temperature dependence of τ.

Supplementary References

[1] Gradshteĭn, I. S., Ryzhik, I. M. and Jeffrey, A. Table of integrals, series, and products. (Academic Press, 2007).
[2] Isichenko, M. B. Percolation, statistical topography, and transport in random media. *Rev. Mod. Phys.* **64**, 961-1043 (1992).
[3] Gallagher, P. *et al.* Quantum-critical conductivity of the Dirac fluid in graphene. *Science* **364**, 158-162 (2019).
[4] Xin, N. *et al.* Giant magnetoresistance of Dirac plasma in high-mobility graphene. *Nature* **616**, 270–274 (2023).
[5] Fritz, L., Schmalian, J., Müller, M. and Sachdev, S. Quantum critical transport in clean graphene. *Phys. Rev. B* **78**, 085416 (2008).
[6] Ralph A. Höpfel, Jagdeep Shah, Peter A. Wolff, and Arthur C. Gossard Phys. Rev. Lett. **56**, 2736 (1986)
[7] Luyi Yang, J. D. Koralek, J. Orenstein, D. R. Tibbetts, J. L. Reno, and M. P. Lilly Phys. Rev. Lett. **106**, 247401 (2011)
[8] *Semiconductors*, edited by R. A. Smith (Cambridge University Press, New York, 1978)
